# Replacing Ecologically Risky Trifluoroacetic Acid and Acetonitrile With Methanesulfonic Acid and Dimethyl Carbonate in High‐Performance Liquid Chromatography Analyses of Small Molecule Drugs

**DOI:** 10.1002/jssc.70425

**Published:** 2026-05-05

**Authors:** Franziska Pögel neé Steinicke, Taha B. El‐Jourani, Jana Haegner, Matthias Schiedel, Hermann Wätzig

**Affiliations:** ^1^ Institute of Medicinal and Pharmaceutical Chemistry Technische Universität Braunschweig Braunschweig Germany; ^2^ Institut für Pharmazeutische und Medizinische Chemie Universität Münster Münster Germany; ^3^ Center of Pharmaceutical Engineering Technische Universität Braunschweig Braunschweig Germany

**Keywords:** chromatography, forever chemicals, PFAS, purity analysis, sustainability

## Abstract

It has long been recognised that, in the long term, hazardous acetonitrile (ACN) as a mobile‐phase component and the environmentally critical trifluoroacetic acid (TFA) as an ion‐pairing reagent should be replaced in high‐performance liquid chromatography (HPLC). Nevertheless, methods relying on precisely these reagents are still widely used. Herein, we show that alternative approaches for the analysis of small molecule drugs are readily applicable, using examples from pharmaceutical quality control and medicinal chemistry. First, the principles of green analytical chemistry were implemented in an alternative HPLC method for the theophylline monohydrate monograph of the European Pharmacopoeia, without altering the core methodology. Among the various conditions tested, the use of 2% dimethyl carbonate (DMC) provided equivalent separation and an even slightly improved resolution compared with the original ACN‐based pharmacopoeial method. In a second approach, we present a highly sustainable gradient HPLC method for routine purity analysis of synthesis products by replacing TFA with safe, biodegradable, and environmentally benign methanesulfonic acid (MSA). Furthermore, in the case of this gradient method, ACN could be replaced by the biodegradable and environmentally friendly DMC. An eluent composition consisting of 42 parts DMC, 23 parts EtOH, 35 parts H_2_O and 0.1 parts MSA was found to be equivalent to ACN containing 0.1% TFA. By employing a reproducible protocol using EtOH as a co‐solvent, we were able to overcome current challenges associated with the use of organic carbonates in HPLC, which primarily arise from their limited miscibility with water. Given the continued widespread use of gradient HPLC methods employing ACN as an eluent and TFA as an acidic modifier, the methods presented herein offer significant potential to advance the implementation of sustainability in pharmaceutical quality control and medicinal chemistry.

## 1 Introduction

High‐performance liquid chromatography (HPLC) is an analytical separation technique to identify and quantify specific components in complex mixtures originating from food, chemical, biological, environmental and agricultural sources. Moreover, HPLC also remains an indispensable tool in medicinal chemistry as well as pharmaceutical research and qualitysequence control.

Acetonitrile (ACN) and trifluoroacetic acid (TFA) are widely used in analytical and preparative HPLC [1, 2, 3, 4, 5, 6, 7, 8, 9, 10]. For example, HPLC gradient methods employing these two reagents are routinely used to assess the purity of newly synthesised druglike molecules. Approximately 1100 monographs of the European Pharmacopoeia (Ph. Eur.) include ACN in HPLC‐based purity and potency testing, respectively. Yet, the environmental and health impacts associated with toxic eluents and modifiers highlight the urgent need for more acceptable alternatives.

In the following, the hazardous qualities of most organic modifiers that are used in research and quality control are discussed. For example, ACN shows toxicity through inhalation, ingestion and skin absorption. In addition, security of supply is problematic for ACN, as the availability fluctuates greatly as a by‐product of other industries, such as the paint industry. In recent years, the price has risen continuously at an above‐average rate [11]. Several less‐toxic alternatives to ACN have been discussed in recent years, including organic carbonates (i.e. dimethyl carbonate [DMC] and propyl carbonate [PC]) and ethanol (EtOH).

DMC is a moderately polar, flammable organic solvent with no known mutagenic or irritating effects [1]. Its eluotropic strength is more than twice that of ACN and five times higher than that of methanol (MeOH) [12]. Consequently, the major advantages of DMC are its low toxicity and the reduced solvent consumption required due to its high elution strength. A further advantageous but also limiting property of DMC is its degradability. In aqueous environments, DMC undergoes hydrolysis to MeOH and carbon dioxide, meaning that it is not a persistent chemical. However, this also limits the long‐term stability and storability of aqueous DMC solutions [13]. PC, a cyclic organic carbonate, has been discussed as an alternative HPLC solvent. Compared to DMC, PC exhibits higher viscosity and lower elution strength. In our experience, the differences between the two solvents are minor.

Ethanol is a widely used and highly accepted sustainable mobile phase constituent [14, 15]. It is significantly less toxic than other traditional HPLC solvents such as ACN and MeOH [16]. Beyond its use as a standard solvent in HPLC analyses, EtOH can be employed as a co‐solvent or solubiliser to prevent phase separation between otherwise immiscible solvents [17]. The acute toxicity of EtOH is frequently discussed; however, this is largely in the context of its often abusive consumption in large gram quantities. Milligram quantities can certainly be considered safe. Moreover, increasing production of ‘bioethanol’ is expected to further reduce costs, enhancing its attractiveness as a sustainable solvent option.

TFA is widely used in HPLC as an effective ion‐pairing reagent and is a strong organic acid with a p*K*_a_ of 0.43 that exists predominantly in its dissociated form at pH values above 0.43 [18]. Owing to its trifluoromethyl group, TFA belongs to the class of per‐ and polyfluoroalkyl substances (PFAS). This perfluorination renders TFA highly chemically inert [19], resulting in extremely slow degradation and biodegradation. Consequently, PFAS and especially TFA are often referred to as “forever chemicals”. Notably, TFA is currently the most prevalent PFAS found in the environment [20]. The European Chemicals Agency (ECHA) has linked TFA and its salts to reproductive toxicity [19]. At the same time, the environmental impact of TFA remains incompletely understood, fuelling debate over its ecological risks considering its persistence, global ubiquity, and steadily rising concentrations. For example, a continuous increase in TFA concentrations has been observed in vintage wines since the 1940s, with a drastic increase over the past two decades [21, 22]. The lack of biodegradability represents a major concern and is a key reason why TFA has been targeted for replacement by more sustainable and environmentally benign alternatives. In this context, it is noteworthy that in 2024, five countries submitted a proposal to the ECHA advocating for a comprehensive ban on PFAS, further underscoring the urgency of identifying viable substitutes for TFA [23].

Due to the aforementioned drawbacks of TFA as an ion‐pairing reagent, several alternatives have been discussed over the recent years. For our studies, we focused on methane sulfonic acid (MSA) as an alternative ion‐pairing reagent, as it is already known within the analytical and pharmaceutical community [24, 25]. MSA has a p*K*_a_ of approximately ‐1.9 and is therefore an even stronger organic acid than TFA. Importantly, MSA exhibits superior properties in terms of storage stability (room temperature) and, most notably, its biodegradability. It is considered readily biodegradable (OECD 301D closed bottle, etc.) and ultimately decomposes into carbon dioxide and sulphate [24]. In aqueous environments, MSA and TFA exhibit comparable acid strengths and very similar ion‐pairing properties [26]. Another advantage of MSA over TFA is its significantly lower corrosiveness [25]. This aspect is particularly significant when considering the costs associated with TFA‐induced corrosion damage to HPLC pumps and other stainless‐steel HPLC components. The longer service lifetimes and reduced downtime expected from replacing TFA add an additional dimension of economic efficiency and sustainability to methods that avoid the use of TFA. In addition to its comparable ion‐pairing properties and significantly improved sustainability profile, MSA is less toxic and therefore considerably safer than TFA. More than 20 MSA salts of structurally diverse basic drugs (referred to as mesylates) are approved pharmaceuticals, including saquinavir mesylate (HIV treatment), paroxetine mesylate (depression), eprosartan mesylate and amlodipine mesylate (both for hypertension) [27]. The approval of multiple mesylate drugs, even for long‐term treatment of chronic diseases, compellingly highlights the safety of MSA for human use.

**FIGURE 1:**
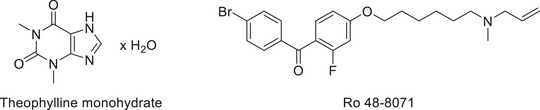
Chemical structures of the monographed drug substance, theophylline monohydrate and the experimental drug candidate Ro 48–8071.

Sustainability is increasingly recognised as a key objective across all scientific disciplines, including the HPLC purity analyses of small molecules in analytical and pharmaceutical chemistry. Accordingly, the aim of this study was to demonstrate the straightforward transition from long‐established ACN‐based HPLC methods to alternative approaches employing more sustainable solvents, particularly DMC. A second major goal of this study was to show that the environmentally critical TFA, which is still widely regarded as the gold‐standard ion‐pairing reagent and pH modifier, can be readily replaced with MSA without compromising analytical performance. To demonstrate the feasibility of these approaches for both isocratic and gradient methods, theophylline monohydrate [28] was selected as a representative monographed drug substance analysed using an isocratic method according to the Ph. Eur., whereas Ro 48–8071 [29] served as an example of an experimental drug candidate evaluated by means of a gradient method (Figure 1).

## 2 Methods and Materials

### 2.1 Instruments

The LC system used was a VWR Hitachi Chromaster with a quaternary gradient pump, column oven, DAD and a thermostat autosampler.

### 2.2 Chemicals and Materials

MeOH, EtOH absolute 99.8%, ACN 99.8% and propan‐2‐ol (*i*‐PrOH) 99.5% were HPLC grade (Fisher Chemical, UK). The DMC was of 99% purity (abcr, Karlsruhe, Germany) and the propylene carbonate (PC) of 99.95% (BLD‐Pharm, Shanghai, China). The MSA was of 99% HPLC grade (TCI, Tokyo, Japan), and the TFA contained 99.9% (abcr, Karlsruhe, Germany). The column used in the experiments was a Reprosil XR 120 C18 5 µm with an inner diameter of 4.6 µm and a length of 150 mm. 3‐Methyl‐3,7‐dihydro‐1*H*‐purin‐2,6‐dione; (3‐methylxanthine) 98% as impurity B was purchased from TCI Tokyo, Japan and *N*‐(6‐amino‐1,3‐dimethyl‐2,4‐dioxo‐1,2,3,4‐tetrahydropyrimidin‐5‐yl)formamide 95% as impurity C from BLD Pharm Shanghai, China. Theophylline monohydrate, theobromine and caffeine were of pharmaceutical standard (Figure 3). Impurity D (*N*‐methyl‐5‐(methylamino)‐1*H*‐imidazol‐4‐carboxamide; theophyllidine) was prepared by hydrolysing theophylline (see Supporting Information). Ro 48–8071 (Figure 1) was synthesised according to Morand et‐al., [29]. The ultrapure water was provided by a Sartorius Arium Pro system (Göttingen, Germany).

### 2.3 Isocratic Method

The following method was taken from the Ph. Eur. instructions for testing the purity of theophylline monohydrate. The only difference is the dimensions of the column [28].

The original mobile phase of the Ph. Eur. consists of 7% ACN and 93% buffer (NaOAc 1.36 g/L, HOAc 99% 5 mL/L). The second tested mobile phase consists of 2% DMC and 98% buffer. The other three mobile phases tested consist of 3% PC and 97% buffer, 10% EtOH and 90% buffer and 4% EtOH with 3% *i*‐PrOH and 93% buffer.

The flow rate was set to 2 mL/min, and the detection wavelength was 272 nm. The temperature of the column oven was adjusted to 20°C, and the injection volume was 20 µL. The mass concentrations of the analytes were 0.01 mg/mL in each case. All test solutions were prepared with their mobile phase accordingly.

Mobile phases with DMC and PC need to be mixed before use (ultrasonic bath), because although they are soluble in this concentration, they exhibit limited miscibility (kinetic solubility) with water.

### 2.4 Gradient Method

The flow rate was set to 0.5 mL/min, and the detection wavelength was set to 254 nm. The temperature of the column oven was levelled at 25°C, and the injection volume of the analyte was 2.5 µL. The content of Ro 48–8071 was 1.06 mg/mL in MeOH.

The original mobile phases consist of solution A, which contains ACN with 0.1% TFA, and solution B, which contains water with 0.1% TFA. The newly developed mobile phases, which can replace the original mobile phases equivalently, consist of solution A containing DMC/EtOH/H_2_O (42:23:35) with 0.1% MSA and solution B containing H_2_O/EtOH (80:20) with 0.1% MSA.

The gradient is shown in Table 1.

**TABLE 1:** Gradient conditions for the routine purity analysis of newly synthesised substances.

**Time [min]**	**A [%]**	**B [%]**
0–3	10	90
18–24	95	5
27–33/35	10	90

To be able to use such high concentrations of DMC with water in the mobile phase, EtOH needs to be added to both gradient components to avoid phase separation in and outside of the HPLC system. Further explanation in R&D.

## 3 Results and Discussion

### 3.1 Methods Selection Rationale

The ACN‐based isocratic HPLC method for theophylline monohydrate was taken as an example from the Ph. Eur. [28] to demonstrate the versatility of sustainable mobile‐phase alternatives in hydrophilic separation systems. In parallel, a widely used ACN‐ and TFA‐based gradient method for evaluating the purity of synthesis products was selected, due to its broad adoption in medicinal chemistry and the correspondingly high potential impact of newly developed sustainable alternatives.

### 3.2 Isocratic Method

**FIGURE 2:**
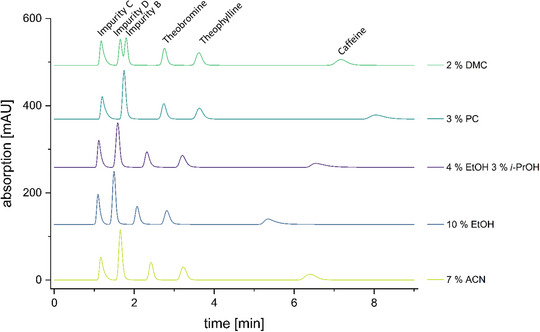
Isocratic method for purity testing of theophylline monohydrate with the impurities B, C, D, caffeine and theobromine (for chemical structures see Figure 3). Displayed are the original acetonitrile (ACN)‐based method and four more sustainable alternatives with comparable or even better peak separation (e.g. dimethyl carbonate [DMC]). The chromatograms are displayed offset, with the baseline starting at zero in each case. Various offsets are selected for appropriate graphical representation. Elution order in all chromatograms: impurities C, D and B (only separated using DMC); theobromine, theophylline and caffeine. See Table 2 for an overview of the detailed mobile phase compositions, observed back pressures, and comments on the individual methods.

First, various alternative mobile‐phase compositions were evaluated in addition to those shown in Figure 2. Initial experiments using EtOH concentrations up to 20% [30] resulted in excessively short retention times and thus insufficient resolution. Stepwise reduction to 10% EtOH yielded acceptable retention behaviour, whereas 7% EtOH produced excessively long retention times and pronounced tailing of the caffeine peak.

**FIGURE 3:**
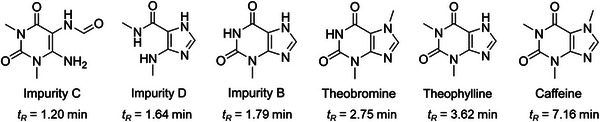
Theophylline and potential impurities were used for the development of the isocratic methods. The compounds are presented in the elution order observed in the 2% dimethyl carbonate (DMC) run, together with their corresponding retention times.

Second, *i*‐PrOH was evaluated. At 7% *i*‐PrOH, retention times were too short, prompting the investigation of mixed solvent systems with *i*‐PrOH and EtOH. To this end, mixtures with EtOH were prepared and tested, starting with 2% *i*‐PrOH and 5% EtOH, resulting in strong tailing and 2% *i*‐PrOH and 4% EtOH, resulting in long retention times. The optimal compromise between suitable retention time, peak shape, and resolution was achieved using a mixture of 4% EtOH and 3% *i*‐PrOH, which closely matched the original ACN‐based chromatograms. For those wishing to rely on well‐established solvents, the combination of EtOH and *i*‐PrOH represents a sustainable alternative, achieving comparable separation quality compared to ACN‐based methods, by using minimal amounts of sustainable, non‐toxic organic solvents.

**FIGURE 4:**
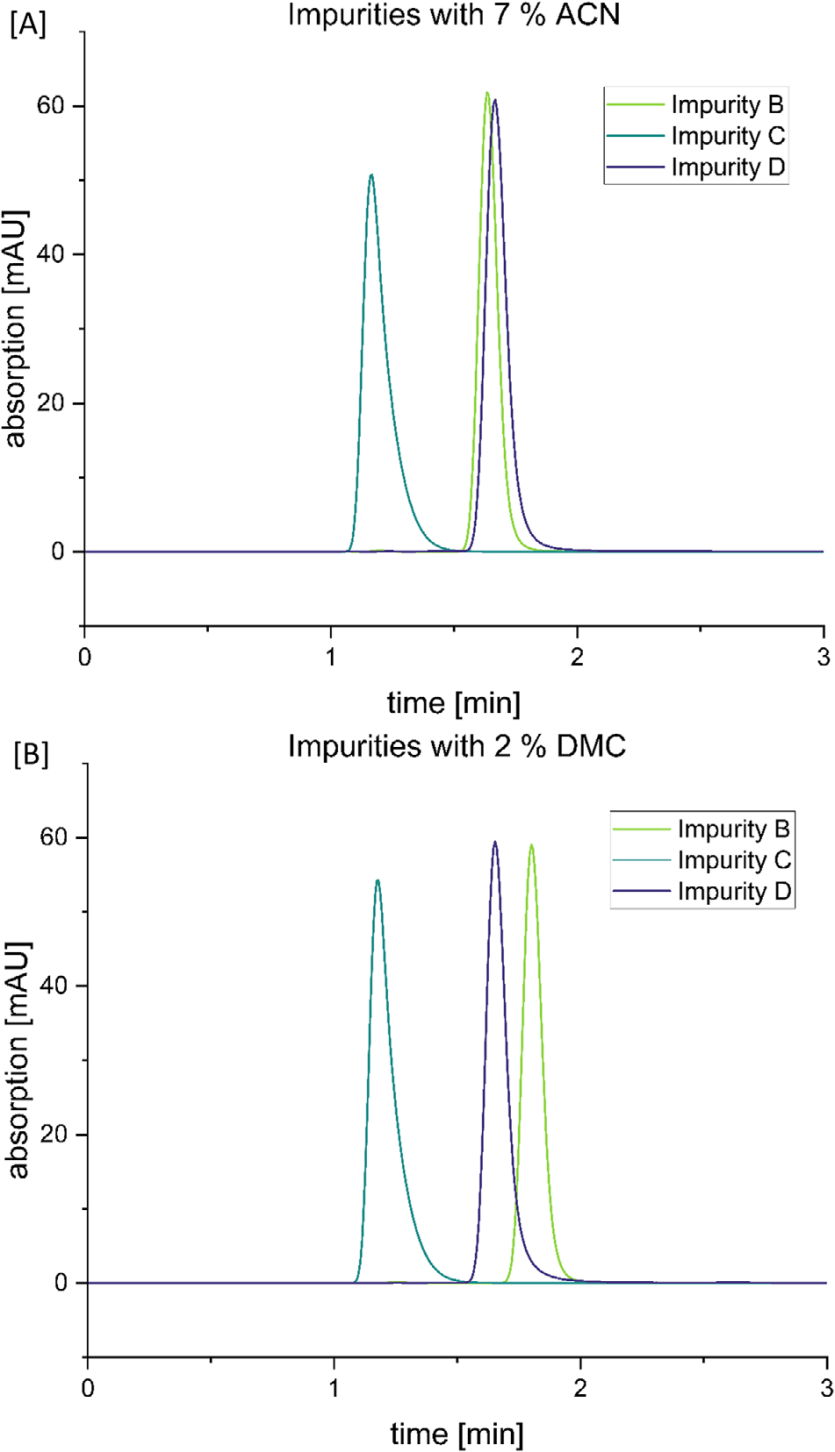
Comparison of the resolution of impurities B and D with the [A] acetonitrile (ACN)‐based and [B] dimethyl carbonate (DMC)‐based method, respectively. The shown traces were recorded in individual runs.

Third, DMC was tested at a concentration of 2% [31] and showed immediately very promising results. The work of Kalisz et al. [31], in which DMC and water were used for the chromatographic separation of theobromine and caffeine in tea, did not need to be further improved, even though we used a buffer instead of water. Application of this method resulted in the best peak separation for impurities D and B (Figures 2 and 4B). Furthermore, the retention times of all peaks closely matched those obtained with the original pharmacopoeial method employing ACN, thereby fulfilling our initial objective. However, when working with DMC, one has to consider the poor miscibility of DMC in water. Thus, the mobile phase needs to be mixed before use in an ultrasonic bath, otherwise DMC acts like oil in water. The solubility of DMC in water is about 155 mg/mL [32].

Last, PC was evaluated as a solvent component for this HPLC method. Due to its lower elution strength compared to DMC, slightly higher concentrations (3%) were required to obtain similar results. Notably, small concentration changes had a pronounced effect on retention times. For example, a PC concentration of 5% resulted in too short retention times and insufficient peak separation. Similar to DMC, ultrasonic pretreatment was necessary to obtain a stable mobile phase.

Overall, all five mobile phases optimised for this isocratic method evoked similar results compared to the original ACN‐based method from the Ph. Eur. (Figure 2) and met the pharmacopoeial requirement of a minimum resolution of 2.0 between the peaks of theobromine and theophylline. The relatively pronounced tailing of the caffeine peak observed with PC (3%), EtOH (10%), and EtOH (4%)/*i*‐PrOH (3%) can be attributed to the significantly higher lipophilicity of caffeine compared to the other test substances. This tailing was accepted to enable adequate separation of the more hydrophilic impurities while still allowing caffeine to be analysed within the same chromatographic run.

The DMC‐based mobile phase showed significantly less tailing of the caffeine peak and, most notably, enabled separation of impurities B and D (Figures 2 and 4B), which co‐eluted under the original ACN‐based method (Figures 2 and 4A). With this, we clearly demonstrate that replacing ACN with less toxic and more sustainable alternatives is straightforward and can result in equivalent or even improved chromatographic performance.

All the alternatives to ACN had one thing in common: they all generate significantly higher back pressure in the HPLC system. The pressures achieved were a maximum of 280 bar at 20% EtOH and 2 mL/min. 7% *i*‐PrOH had 200 bar, 2% DMC and 3% PC had a back pressure of around 160 bar.

Regarding the practical implementation of the new methods, one of the main challenges was the still‐limited understanding of the physicochemical properties of organic carbonates (i.e. DMC and PC). Although these solvents exhibit a defined solubility limit in water, they are not readily miscible even below this threshold and require additional processing to form stable solutions. In this study, ultrasonic treatment proved effective, with a few minutes in an ultrasonic bath being sufficient to achieve homogeneous and stable mobile phases. However, this pretreatment requirement also implies that binary carbonate–water systems cannot be generated in situ using an HPLC gradient mixer and are thus currently best suited for isocratic applications employing pre‐mixed solvent systems. An overview of the composition of the studied mobile phases, observed back pressures, and comments on the individual methods can be found in Table 2.

**TABLE 2:** Comparison of mobile phase properties for isocratic theophylline monohydrate purity testing. Buffer: NaOAc 1.36 g/L, HOAc 99% 5 mL/L.

Mobile phase	Backpressure	Comments
2% DMC 98% buffer [35]	166 bar	Best overall outcome for separation. Mixing before use because of limited kinetic solubility.
3% PC 97% buffer	164 bar	Mixing before use because of limited kinetic solubility.
4% EtOH 3% i‐PrOH 93% buffer	190 bar	Best combination of properties of EtOH and i‐PrOH (separation, peak shape and retention time)
10% EtOH 90% buffer	215 bar	
7% ACN 93% buffer	<80 bar	Original Ph. Eur. method.

### 3.3 Gradient Method

ACN‐based gradient methods employing TFA as an acidic modifier represent standard approaches for purity assessment of synthesis products (see introduction) and are widely used with only minor variations in TFA content. While some gradient methods use TFA solely in the aqueous phase, others contain TFA in both the aqueous and the ACN phases [4, 5, 6, 7, 8, 9, 10]. Owing to their broad adoption, particularly within the medicinal chemistry community, these ACN‐ and TFA‐based gradient methods provide an ideal framework for demonstrating the feasibility of more sustainable alternatives and for promoting the implementation of sustainability in routine laboratory practice.

**FIGURE 5:**
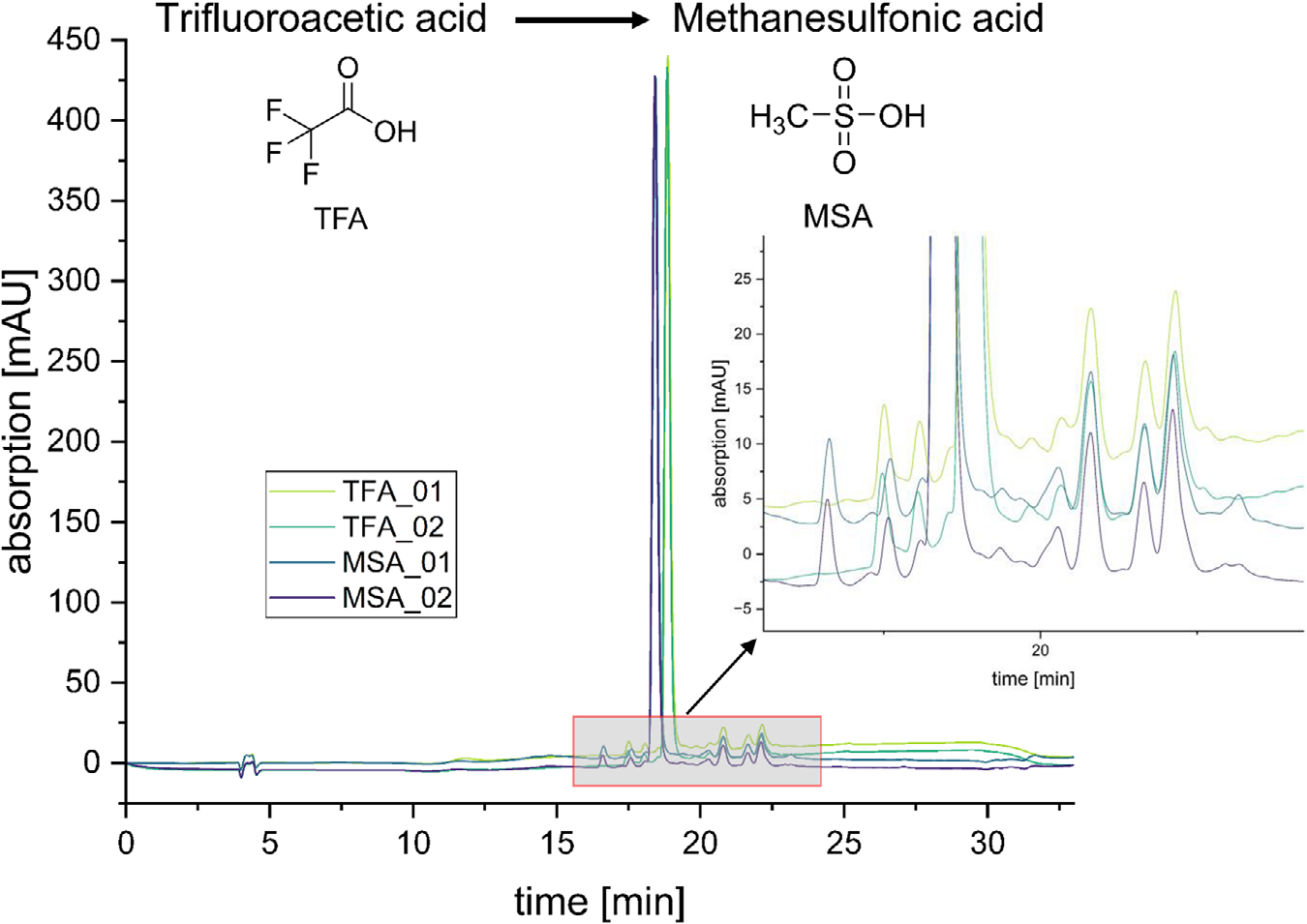
Implementation of methanesulfonic acid (MSA) as a sustainable alternative to trifluoroacetic acid (TFA) as an ion‐pairing reagent. The figure shows the high‐performance liquid chromatography (HPLC) chromatograms obtained using an acetonitrile (ACN)‐based gradient method for the test compound Ro 48–8071, employing 0.1% TFA (light and dark green) or 0.1% MSA (blue and purple) as the ion‐pairing reagent. The right‐hand inset presents a magnified view of the major peaks and minor impurities.

First, TFA was replaced by MSA without any other changes to the underlying core methodology. Using a re‐synthesised batch of the oxidosqualene cyclase inhibitor Ro 48–8071 [29] as a representative test sample for newly synthesised druglike molecules, nearly identical retention times for the target compound and at least equivalent chromatographic separation of the minor impurities were obtained. Repeated runs indicated high reproducibility of the new method. These results demonstrate full substitutability of TFA by MSA, with comparable or even improved chromatographic performance (Figure 5). This result underscores that the hazardous, corrosive, and environmentally highly problematic TFA can be readily replaced as an ion‐pairing reagent by the safe, less corrosive and biodegradable MSA in a straightforward manner.

**FIGURE 6:**
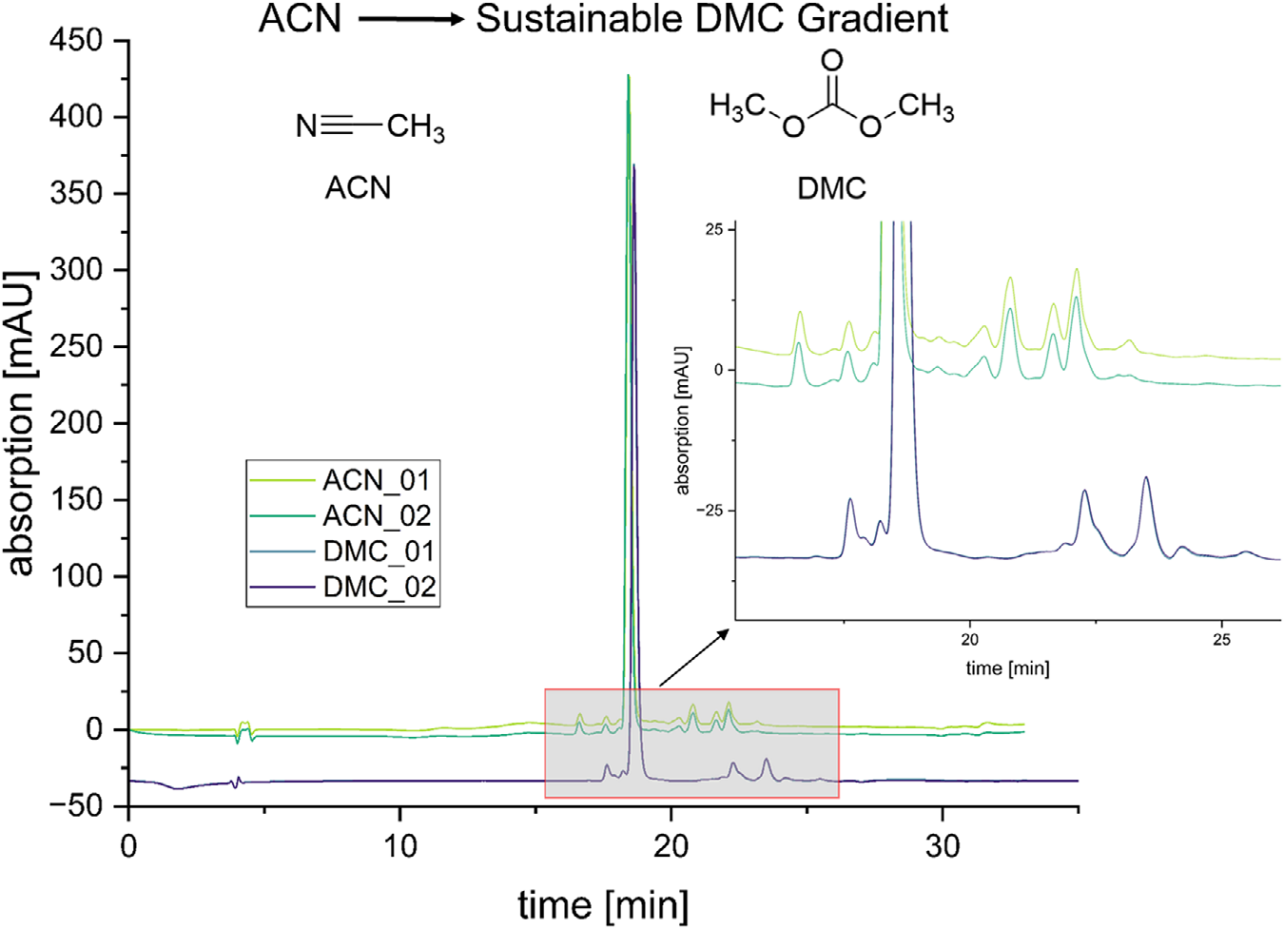
Implementation of DCM as a sustainable alternative to acetonitrile (ACN) as a high‐performance liquid chromatography (HPLC) eluent. The figure shows HPLC chromatograms obtained using either an ACN‐based (light and dark green) or a DCM‐based gradient method (blue and purple; note that both traces overlap and therefore appear as a single trace). In all cases, 0.1% methanesulfonic acid (MSA) was used as an ion‐pairing reagent. Details of the eluent compositions and gradient conditions are provided in section 2.4 and Table 1, respectively.

Second, to enable the use of DMC as a mobile‐phase component in gradient elution and thereby exploit its previously discussed advantages, it was first necessary to address the solubility limitations of binary DMC–water mixtures. EtOH has proven to be a helpful cosolvent and allows DMC and water to be easily used together. However, the reported mixing ratio could not be reproduced [17], which is why a customised mixing protocol was developed. This resulted in a composition of DMC/EtOH/H_2_O/MSA (42:23:35:0.1), which was found to be equivalent to ACN with 0.1% TFA. To prevent phase separation of this DMC‐based, predominantly organic phase during gradient operation with an aqueous phase, the addition of a defined amount of EtOH to the aqueous phase was required. Preliminary investigations to determine the lowest possible proportion of EtOH as a cosolvent in the aqueous phase yielded a concentration of 20%. By using these compositions for the primarily organic phase (DMC/EtOH/H_2_O/MSA [42:23:35:0.1]) and the primarily aqueous phase (H_2_O/EtOH/MSA [80:20:0.1]), we performed a gradient elution with Ro 48‐8071 as test sample. Employing the same A/B gradient ramp as that used in the benchmark ACN‐ and TFA‐based method (see materials and methods 2.4, Table 1) with our newly composed sustainable eluents, we obtained almost identical retention times for Ro 48‐8071 and similar chromatographic separation of the minor impurities (Figure 6). Again, repeated runs indicated high reproducibility of the new method. With these results, we show that both TFA and ACN can be replaced by more sustainable alternatives without compromising analytical performance.

## 4 Conclusions and Outlook

HPLC methods replacing hazardous and environmentally harmful substances such as ACN and TFA with more sustainable and less toxic alternatives were successfully developed, resulting in equivalent or even improved chromatographic performance. In particular, DMC and methanesulfonic acid (MSA) proved to be fully viable substitutes.

Mobile phases with low amounts of DMC (e.g. 2%) are readily implemented for isocratic methods, but require ultrasound pretreatment to facilitate the mixing process. Higher amounts of DMC for more hydrophobic mobile phases are best implemented using mixtures of DMC and EtOH as a co‐solvent to overcome the limited aqueous solubility of DMC. A composition of DMC/EtOH/H_2_O (42:23:35) was found to be equivalent to 100% ACN in terms of elution strength and chromatographic performance. This primarily organic phase can be used in combination with an aqueous phase (H_2_O/EtOH [80:20]) for gradient elution. In general, the use of EtOH as a cosolvent was the key to significantly broadening the applicability of organic carbonates as mobile phase constituents.

As an acidic modifier for ion‐pairing, the hazardous, corrosive and environmentally highly problematic TFA can be readily replaced as an ion‐pairing reagent by the safe, less corrosive, and biodegradable MSA in a straightforward manner. MSA can be employed as a sustainable alternative to TFA not only in conventional ACN‐based gradient methods but, most importantly, also in the newly developed DMC‐based gradient method, resulting in a highly sustainable gradient approach with broad applicability for purity analysis across a wide range of test substances. Notably, the newly developed gradient system can be operated using the same A/B gradient ramp as the original ACN‐ and TFA‐based method, yielding nearly identical retention times and equivalent peak separation.

In light of the at least equivalent chromatographic performance of DMC as an eluent and MSA as an ion‐pairing reagent, together with the continued widespread use of ACN‐ and TFA‐based gradient HPLC methods, the approaches presented herein offer substantial potential to advance the implementation of sustainability in pharmaceutical quality control and in routine purity analysis in medicinal chemistry.

## Author Contributions

**Franziska Pögel neé Steinicke**: conceptualisation, methodology, validation, investigation, writing – review and editing, writing – original draft and data curation. **Taha El‐Jourani**: investigation, methodology, validation, visualisation, writing review and editing and data curation. **Jana Haegner**: investigation, validation, visualisation, writing – review and editing and data curation. **Matthias Schiedel**: conceptualisation, writing – review and editing, supervision and investigation. **Hermann Wätzig**: conceptualisation, methodology, writing – original draft, writing – review and editing, supervision and investigation.

## Conflicts of Interest

The authors declare no conflicts of interest.

## Supporting information




**Supporting File 1**: jssc70425‐sup‐0001‐SuppMat.pdf.

## Data Availability

The data that support the findings of this study are available from the corresponding author upon reasonable request.
