# Supplementary material for: Replacing Ecologically Risky Trifluoroacetic Acid and Acetonitrile With Methanesulfonic Acid and Dimethyl Carbonate in High‐Performance Liquid Chromatography Analyses of Small Molecule Drugs
Source: J Sep Sci. 2026 May 5;49:e70425. doi: 10.1002/jssc.70425 (PMC13146114; doi:10.1002/jssc.70425)
Supplement: Supplementary file 1 — Supporting File 1: jssc70425‐sup‐0001‐SuppMat.pdf. [file JSSC-49-e70425-s001.pdf]

Supporting Information
Replacing ecologically risky trifluoroacetic acid and acetonitrile with methanesulfonic acid and dimethyl carbonate in HPLC analyses

Franziska Pögel neé Steinicke,[a] Taha B. El-Jourani,[a] Jana Haegner,[a] Matthias Schiedel,[a,b,c]* Hermann Wätzig[a,c]*

[a] Institute of Medicinal and Pharmaceutical Chemistry, Technische Universität Braunschweig, D-38106 Braunschweig, Germany

[b] Universität Münster, Institut für Pharmazeutische und Medizinische Chemie, Corrensstraße 48, D-48149 Münster, Germany

[c] Center of Pharmaceutical Engineering, Technische Universität Braunschweig, Franz-Liszt-Straße 35a, D-38106 Braunschweig, Germany

*Correspondence: matthias.schiedel@uni-muenster.de, h.waetzig@tu-braunschweig.de

Abstract: It has long been recognized that, in the long term, hazardous acetonitrile as a mobile-phase component and the environmentally critical trifluoroacetic acid (TFA) as an ion-pairing reagent should be replaced in HPLC. Nevertheless, methods relying on precisely these reagents are still widely used. Herein, we show that alternative approaches are readily applicable, using examples from pharmaceutical quality control and medicinal chemistry.

First, the principles of green analytical chemistry (GAC) were implemented in an alternative HPLC method for the theophylline monograph of the European Pharmacopoeia (Ph. Eur.), without altering the core methodology. Among the various conditions tested, the use of 2% dimethyl carbonate (DMC) provided equivalent separation and an even slightly improved resolution compared with the original acetonitrile-based pharmacopeial method.

In a second approach, we present a highly sustainable gradient HPLC method for routine purity analysis of synthesis products by replacing TFA with safe, biodegradable, and environmentally benign methanesulfonic acid (MSA). Also, in the case of this gradient method, acetonitrile could be replaced by the biodegradable and environmentally friendly DMC. An eluent composition consisting of 42 parts DMC, 23 parts EtOH, 35 parts H_2O and 0.1 parts MSA was found to be equivalent to ACN containing 0.1% TFA. By employing a reproducible protocol using EtOH as a co-solvent, we were able to overcome current challenges associated with the use of organic carbonates in HPLC, which primarily arise from their limited miscibility with water.

Given the continued widespread use of gradient HPLC methods employing acetonitrile as an eluent and TFA as an acidic modifier, the methods presented herein offer significant potential to advance the implementation of sustainability in pharmaceutical quality control and medicinal chemistry.

Table of Contents

Page	Contents
S3	Supplementary Synthetic Procedures
S5	Supplementary NMR Spectra
S10	Supplementary HPLC Chromatograms
S11	Supplementary References

Supplementary Synthetic Procedures

General remarks: Starting materials were purchased from commercial suppliers (abcr, Alfa Aesar, BLDpharm, TCI, Thermo scientific, Sigma Aldrich) and used without further purification. Solvents were used in p.a. quality and dried according to common procedures, if necessary. Literature known compounds were synthesized according to previously published procedures (Ro 48-8071) [1]. Thin-layer chromatography (TLC) for reaction monitoring was performed with polyester sheets coated with Macherey-Nagel silica gel 60 F_{254} (layer thickness: 0.2 mm) and analysed under UV-light (254 nm). Low resolution mass spectrometry (LRMS) was performed with an Advion expression® compact mass spectrometer (CMS) coupled with a Plate Express® (Advion) TLC plate reader using electrospray ionization (ESI) and atmospheric pressure chemical ionization (APCI). Flash column chromatography was performed with hand packed silica columns 60 M (0.040-0.063 mm, 230-400 mesh) as a stationary phase on a Biotage Selekt automated flash purification system equipped with a diode array detector (DAD). Yields were not optimized. NMR-spectra were recorded with a Bruker Avance 600 (^{1}H: 600 MHz, ^{13}C: 151 MHz) instrument. The spectra are referenced against the NMR solvent or tetramethylsilane (TMS) and are reported as follows: ^{1}H: chemical shift δ (ppm), multiplicity (s = singlet, d = doublet, dd = doublet of doublets, t = triplet, q = quartet, p = quintet, m = multiplet), integration, coupling constant (*J* in Hz). ^{13}C: chemical shift δ (ppm), multiplicity (s = singlet, d = doublet), coupling constant (*J* in Hz). The assignment resulted from HMBC and HSQC experiments. Purity was determined by HPLC and UV-detection and was ≥ 95%. HPLC analyses were performed using a VWR Hitachi Chromaster HPLC system utilizing a DAD (detection at 220 and 254 nm) and a Merck, LiChrospher 100 RP-18 (5µm) column with a flow rate of 0.5 $mL \cdot min^{-1}$. The indicated purity was determined at a wavelength of 254 nm. All tested compounds were dissolved in methanol (M1) before injection. As solvent system the following binary solvent system was used. M1: Elution was performed at room temperature under gradient conditions. Eluent A was water containing 0.1% (v/v) TFA; eluent B was acetonitrile containing 0.1% (v/v) TFA. Linear gradient conditions were as follows: 0-3.0 min: A=90%, B=10%; 3.0-18.0 min: linear increase to A=5%, B=95%; 18.0-24.0 min: A=5%, B=95%, 24.0-27.0 min: linear decrease to A=90%, B=10%; 27.0-30.0 min: A=90%, B=10%.

N-Methyl-4-(methylamino)-1*H*-imidazole-5-carboxamide (theophyllidine)

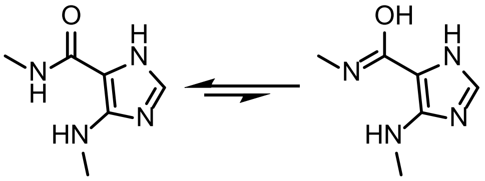

1,3-Dimethyl-7*H*-purin-2,6-dion (500 mg, 2.78 mmol) was suspended in an aqueous sodium hydroxide solution (100 mL, 6 M). The mixture was stirred for 2 h at 90 °C. After complete conversion of the starting material as monitored by TLC the solution was cooled to room temperature and concentrated under reduced pressure until a precipitate was formed. The precipitate was vacuum filtrated, washed with ice-water (2 x 1 mL) and dried under reduced pressure to obtain the crude product. The crude product was purified by flash column chromatography (dichloromethane/methanol: gradient 0-10%) to obtain the product as a beige solid (117 mg, 0.759 mmol, 27%).

R_f = 0.39 (dichloromethane/methanol 20:1);

^{1}H NMR (600 MHz, DMSO-d_6, δ [ppm]): 11.73 (s, 1H, imidazole, H-1, major tautomer), 11.56 (s, 1H, imidazole, H-1, minor tautomer), 7.36 (s, 1H, -OH, minor tautomer), 7.21 (q, *J* = 4.8 Hz, 1H, -NH-imidazole, major tautomer), 7.13 (s, 1H, imidazole, H-2, minor tautomer), 7.09 (s, 1H, imidazole, H-2, major tautomer), 5.98 (q, *J* = 5.5 Hz, 1H, -NH-CO-, major tautomer), 5.66 (m, 1H, -NH-imidazole, minor tautomer), 2.82 (m, 3H, $\underline{H_3}$C-N=COH-, minor tautomer), 2.80 (d, *J* = 5.5 Hz, 3H, $\underline{H_3}$C-NH-CO-, major tautomer), 2.71 (d, *J* = 3.6 Hz, 3H, $\underline{H_3}$C-NH-imidazole, minor tautomer), 2.67 (d, *J* = 4.8 Hz, 3H, $\underline{H_3}$C-NH-imidazole, major tautomer);

^{13}C NMR (151 MHz, DMSO-d_6, δ [ppm]): 165.03 q (C=O, major tautomer), 161.45 q (C-OH, minor tautomer), 153.77 q (imidazole, C-4, minor tautomer), 144.81 q (imidazole, C-4, major tautomer), 133.88 (imidazole, C-2, minor tautomer), 127.11 (imidazole, C-2, major tautomer), 111.35 q (imidazole, C-5, major tautomer), 102.09 q (imidazole, C-5, minor tautomer), 30.82 ($H_3\underline{C}$-NH-imidazole, major tautomer), 29.58 ($H_3\underline{C}$-NH-imidazole, minor tautomer), 24.96 ($H_3\underline{C}$-N=COH-, minor tautomer), 24.85 ($H_3\underline{C}$-NH-CO-, major tautomer);

LRMS (ESI$^+$): *m/z* 177 [M+Na]$^+$; HPLC purity: > 99.9% (M1).

The obtained analytical data are in good agreement with literature values [2].

Supplementary NMR spectra

NMR spectra for compound *N*-methyl-4-(methylamino)-1*H*-imidazole-5-carboxamide (theophyllidine) can be found on the following pages.

^{1}H NMR (600 MHz, DMSO-d_6) for *N*-methyl-4-(methylamino)-1*H*-imidazole-5-carboxamide (theophyllidine)

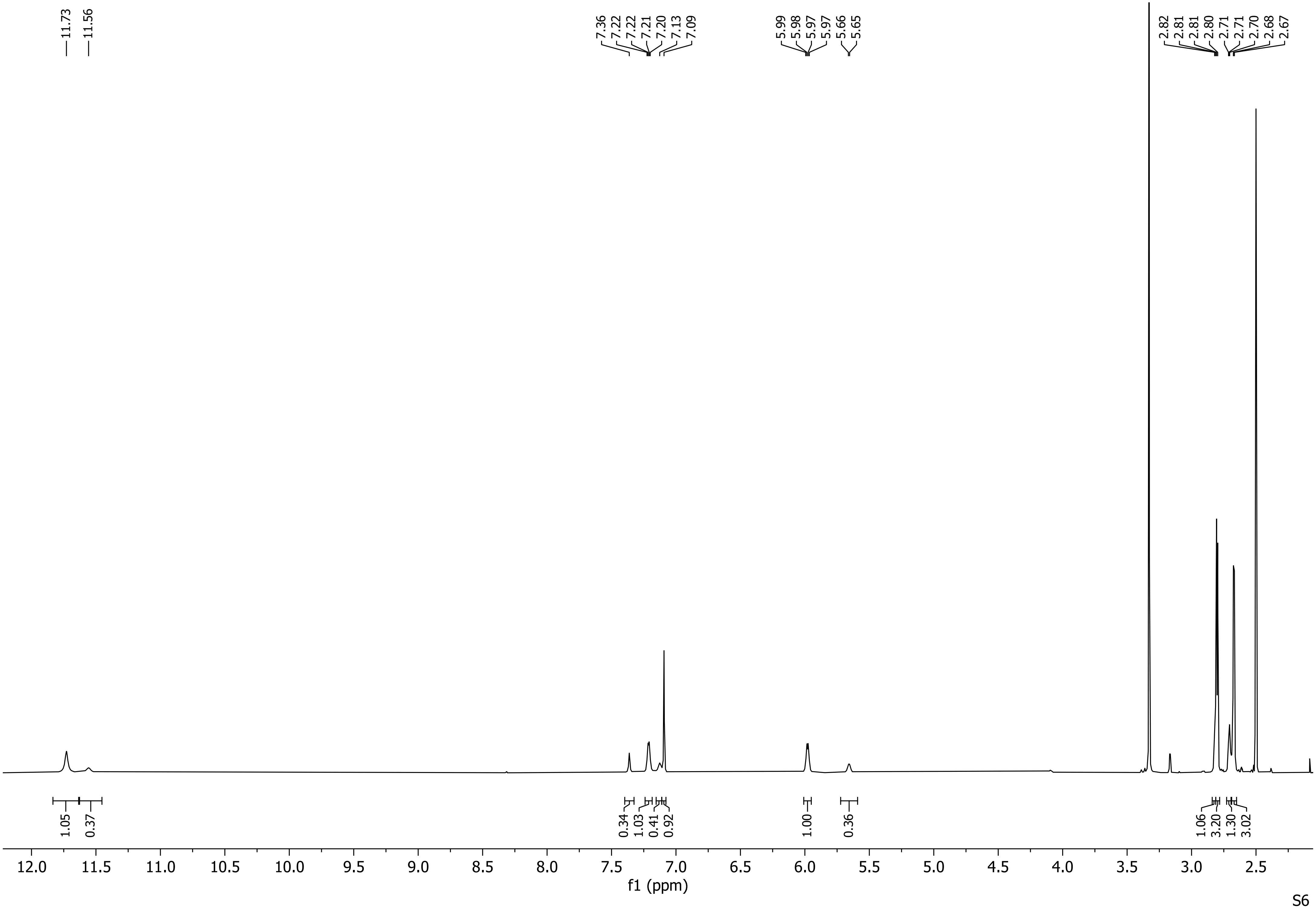

^{13}C NMR (151 MHz, DMSO-d_6) for compound *N*-methyl-4-(methylamino)-1*H*-imidazole-5-carboxamide (theophyllidine)

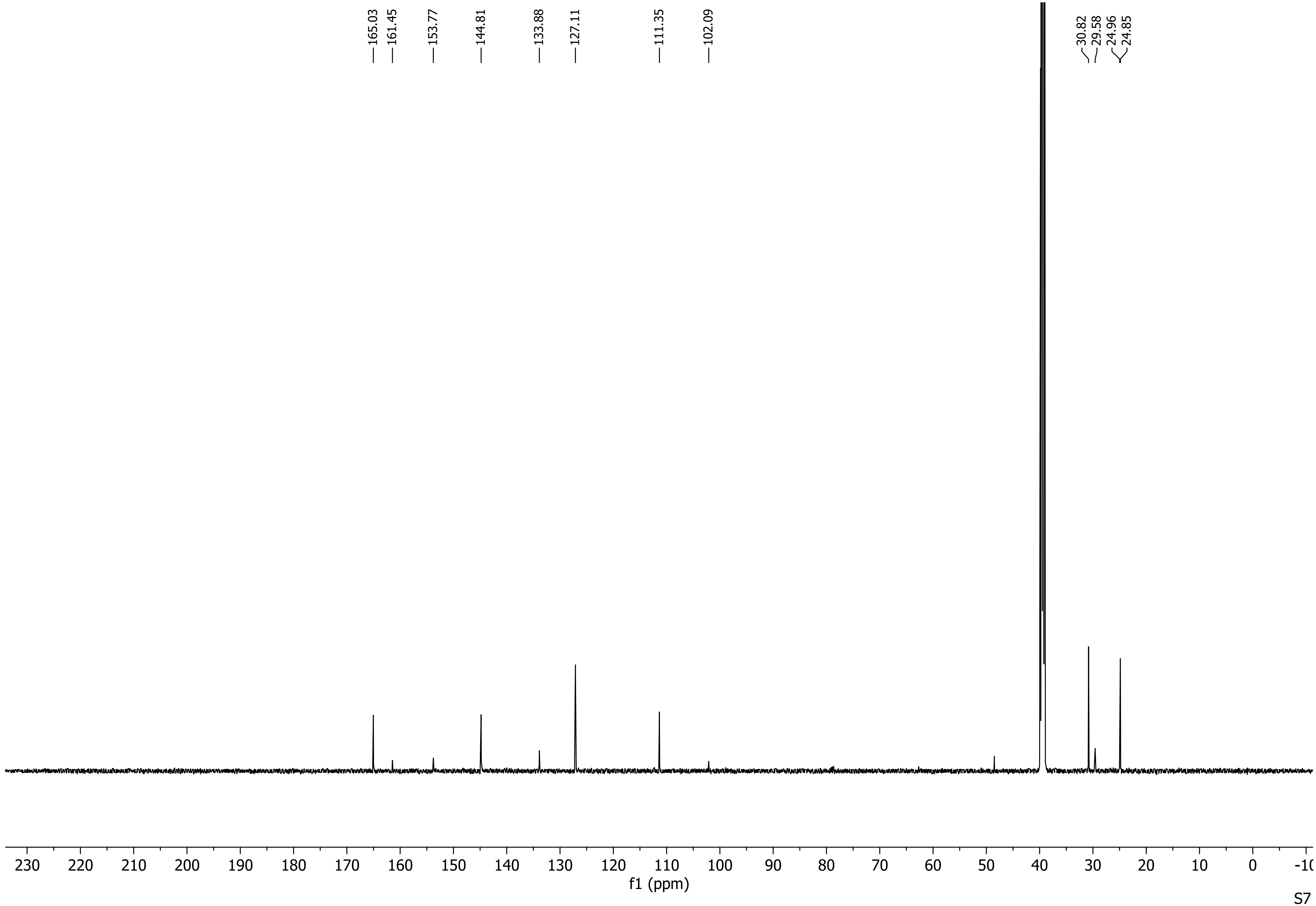

^{13}C NMR (151 MHz, DMSO-d_6) for *N*-methyl-4-(methylamino)-1*H*-imidazole-5-carboxamide (theophyllidine) (zoomed-in view)

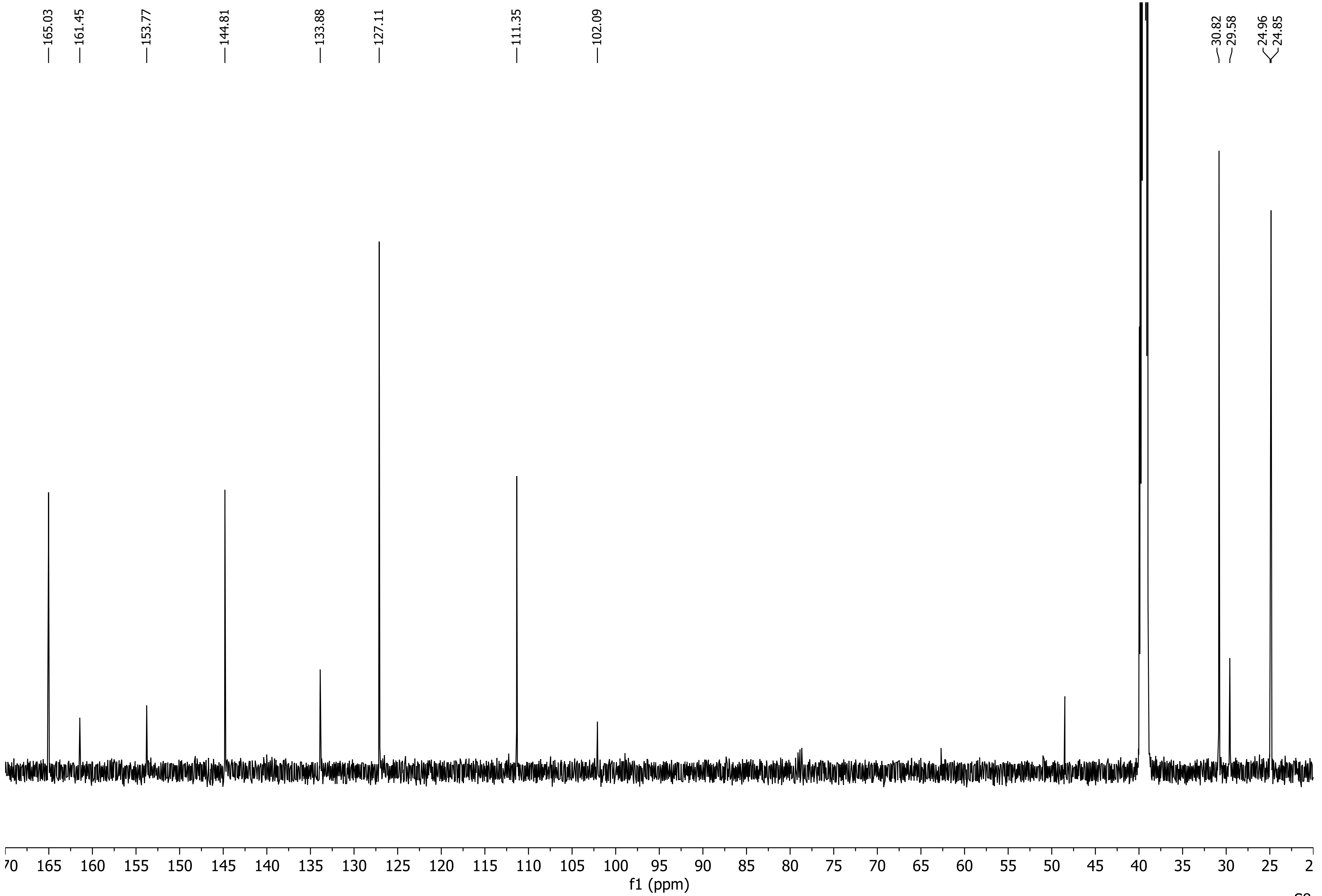

DEPT ^{13}C NMR (151 MHz, DMSO-d_6) for *N*-methyl-4-(methylamino)-1*H*-imidazole-5-carboxamide (theophyllidine) (zoomed-in view)

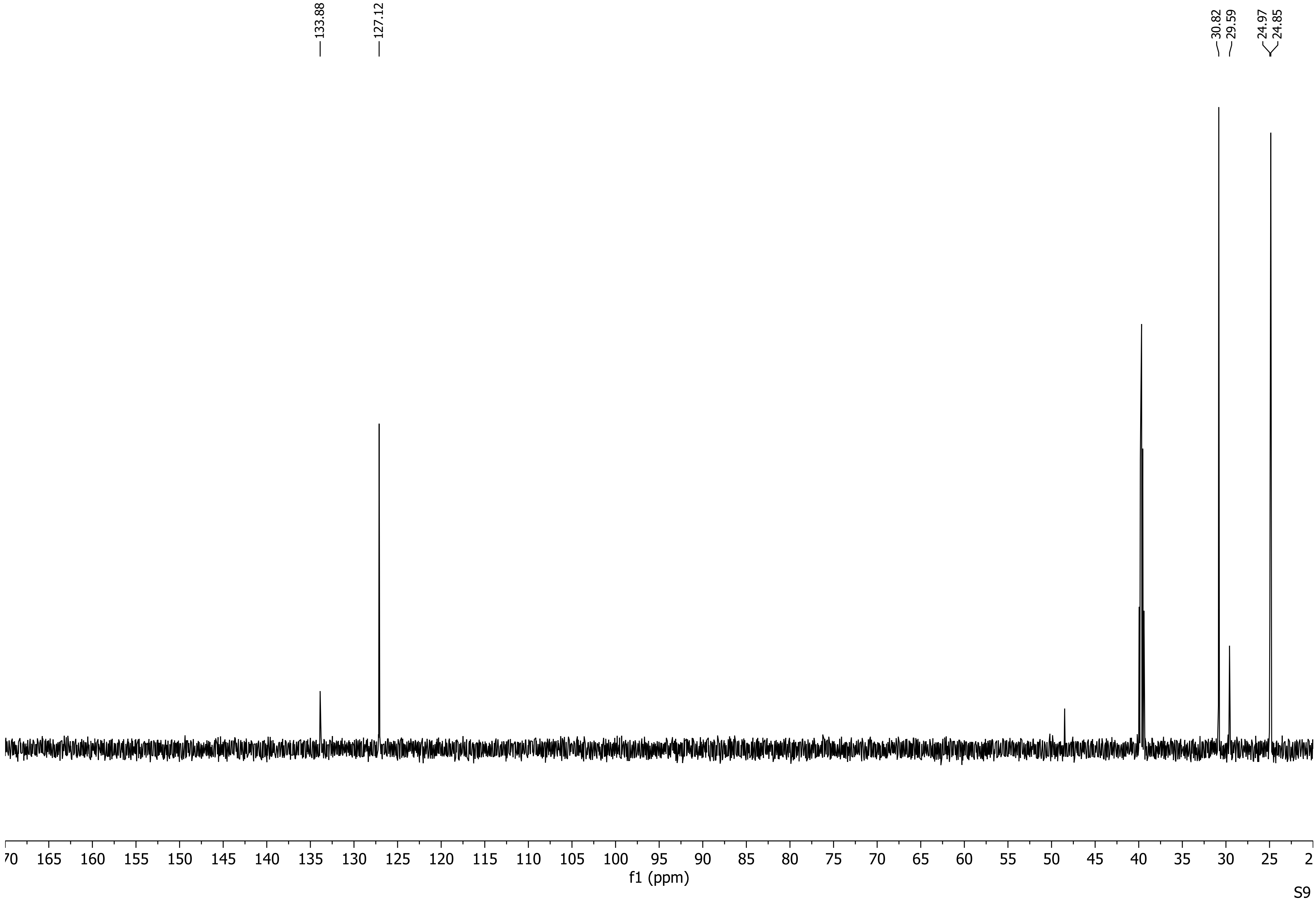

Supplementary HPLC chromatogram

HPLC chromatogram of *N*-methyl-4-(methylamino)-1*H*-imidazole-5-carboxamide (theophyllidine):

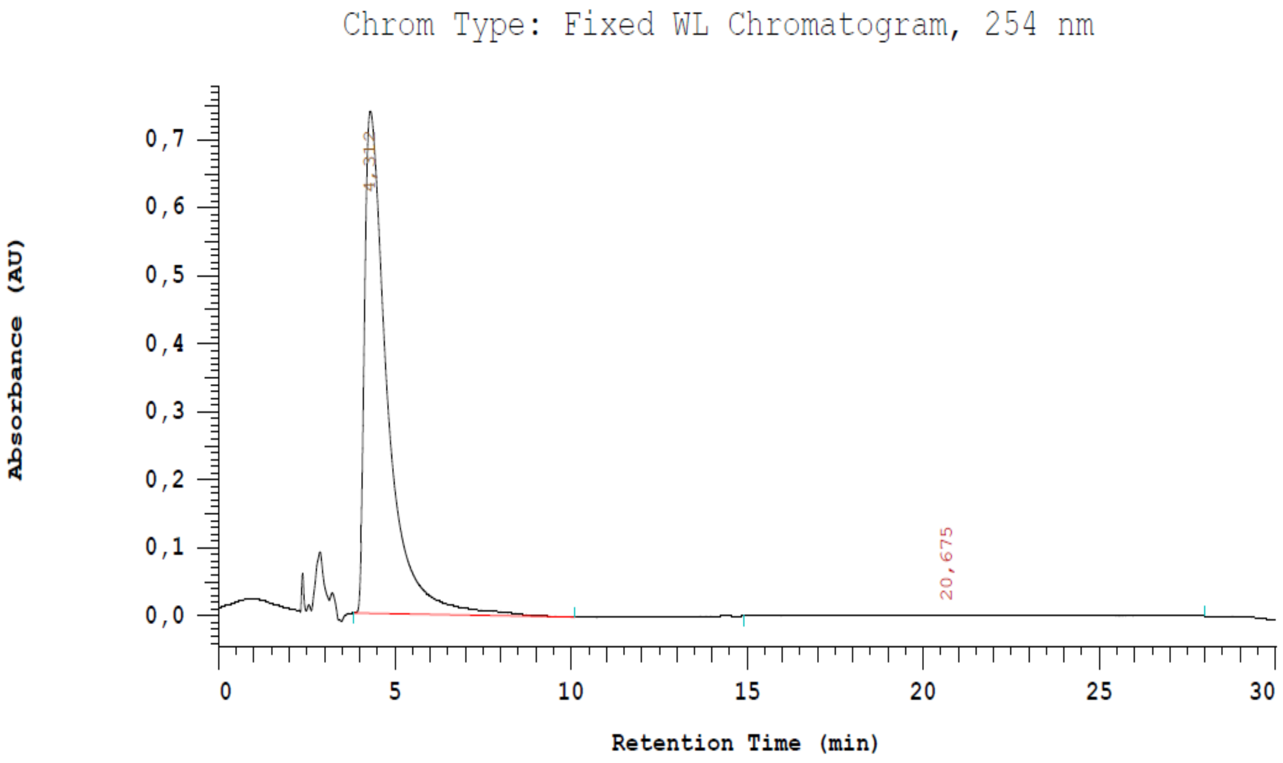

No.	Name	RT	Area	Area %	BC
1		4,312	16558812	100,000	MC
2		20,675	0	0,000	
			16558812	100,000	

Supplementary References

[1] Morand O. H., Aebi J. D., Dehmlow H., Ji Y. H., Gains N., Lengsfeld H., Himber J., Ro 48-8.071, a new 2,3-oxidosqualene:Lanosterol cyclase inhibitor lowering plasma cholesterol in hamsters, squirrel monkeys, and minipigs: Comparison to simvastatin. *Journal of Lipid Research* 1997, 38, 373–390.

[2] Rücker G., Neugebauer M., Heiden P.-G., Isolation of the red dye formed in the theophyllidine reaction. *Archiv der Pharmazie* 1985, 318, 1140–1142.
